# Multimodal carcinoembryonic antigen-targeted fluorescence and radio-guided cytoreductive surgery for peritoneal metastases of colorectal origin: single-arm confirmatory trial

**DOI:** 10.1093/bjsopen/zraf045

**Published:** 2025-04-24

**Authors:** Aaya Darai, Jan Marie de Gooyer, Sander Ubels, Andreas J A Bremers, Philip R de Reuver, Erik H J G Aarntzen, Iris D Nagtegaal, Mark Rijpkema, Johannes H W de Wilt

**Affiliations:** Department of Surgery, Radboud University Medical Centre, Nijmegen, the Netherlands; Department of Surgery, Radboud University Medical Centre, Nijmegen, the Netherlands; Department of Medical Imaging and Nuclear Medicine, Radboud University Medical Centre, Nijmegen, the Netherlands; Department of Surgery, Radboud University Medical Centre, Nijmegen, the Netherlands; Department of Surgery, Radboud University Medical Centre, Nijmegen, the Netherlands; Department of Surgery, Radboud University Medical Centre, Nijmegen, the Netherlands; Department of Medical Imaging and Nuclear Medicine, Radboud University Medical Centre, Nijmegen, the Netherlands; Department of Pathology, Radboud University Medical Centre, Nijmegen, the Netherlands; Department of Medical Imaging and Nuclear Medicine, Radboud University Medical Centre, Nijmegen, the Netherlands; Department of Surgery, Radboud University Medical Centre, Nijmegen, the Netherlands

## Abstract

**Background:**

Selection of suitable candidates for intraoperative tumour detection and cytoreductive surgery (CRS) combined with hyperthermic intraperitoneal chemotherapy (HIPEC) is important for improving outcomes for patients with colorectal peritoneal metastases. Previous research demonstrated the use of single-photon emission computed tomography (SPECT), intraoperative radiodetection, and near-infrared fluorescence (NIRF)-guided surgery with a dual-labelled ^111^In-labelled dodecane tetra-acetic acid (DOTA)–labetuzumab-IRDye800CW tracer to detect peritoneal metastases before operation. The aim of this study was to validate these results.

**Methods:**

A single-centre phase II study was conducted to evaluate the safety and feasibility of ^111^In-labelled DOTA–labetuzumab-IRDye800CW in patients with colorectal peritoneal metastases undergoing CRS-HIPEC. SPECT/computed tomography (CT) was undertaken before surgery, after intravenous administration of 10 mg ^111^In-labelled DOTA–labetuzumab-IRDye800CW (mean 101.25 MBq). During surgery, radiodetection and NIRF imaging were used for tumour detection. Adverse events were assessed, and tumour-to-background ratios (TBRs) and peritoneal cancer index scores were analysed.

**Results:**

Seven patients were included. No study-related severe adverse events were reported. Imaging before surgery revealed previously undetected metastases in one patient. The mean(standard deviation, s.d.) SPECT/CT peritoneal cancer index score was 3(2), and the intraoperative score was 14(7) (*P* = 0.032). A total of 52 lesions were removed during CRS, of which 37 were malignant. With NIRF imaging, 34 (92%) of 37 malignant lesions were detectable. Of 52 fluorescent lesions, 4 were false-positive. Mean(s.d.) fluorescence TBR was 3.4(1.8) and mean radiodetection TBR was 4.4(1.4).

**Conclusion:**

This study confirmed the safety and feasibility of multimodal image-guided surgery in patients with peritoneal metastases.

## Teaser

The trial results indicate that multimodal imaging with ^111^In-labelled dodecane tetra-acetic acid–labetuzumab-IRDye800CW can enhance future cancer treatment by significantly improving tumour detection and surgical precision. This technique allows the intraoperative identification of additional malignant lesions, leading to more thorough resections and potentially altering surgical strategies.

## Introduction

Colorectal cancer may spread to the peritoneal cavity, with peritoneal metastases developing in 3–20% of patients, depending on stage and location of the primary tumour^1,2^. Peritoneal metastatic disease is challenging to treat, as it is often an end-stage disease with a poor prognosis^3,4^. Survival rates began to improve with the introduction of cytoreductive surgery (CRS) combined with hyperthermic intraperitoneal chemotherapy (HIPEC)^5–8^. However, because of the significant morbidity associated with the procedure, it is important to select patients eligible for CRS-HIPEC before surgery^9,10^. CRS-HIPEC should only be conducted in patients with an acceptable peritoneal tumour burden relative to the anticipated improvement in prognosis, as estimated using the peritoneal cancer index (PCI) or other grading methods^10,11^. Conventional imaging techniques such as positron emission tomography or computed tomography (CT) generally underestimate the PCI, so surgeons rely on direct and tactile examinations during surgery to detect additional lesions and to distinguish between benign fibrosis and malignant lesions^12–14^. Enhanced tissue discrimination and higher sensitivity in detection through improved imaging potentially increases the likelihood of achieving complete cytoreduction, which is mandatory for patient survival.

Near-infrared fluorescence (NIRF) imaging techniques using tumour-targeting tracers, such as ^111^In-labelled dodecane tetra-acetic acid (DOTA)–labetuzumab-IRDye800CW^15^, a carcinoembryonic antigen (CEA)-specific tumour-targeted dual-labelled agent, may aid in the intraoperative identification of malignant tissue of colorectal origin^16–18^. Multimodal imaging after administration of a dual-labelled tracer combining a γ-emitting radionuclide and a near-infrared fluorophore on the same targeting molecule may potentially improve cytoreductive surgery^19^. The radionuclide not only enables single-photon emission CT (SPECT)/CT before surgery, but also intraoperative radio-guided differentiation between benign and malignant tissue using a handheld γ probe^20^. The dual-labelled tracer used in this research enables the use of both with a single agent.

In a proof-of-concept phase I dose escalation study, it was demonstrated that the use of ^111^In-labelled DOTA–labetuzumab-IRDye800CW for tumour detection both before and during surgery in 15 patients was safe and identified the optimal dose of tracer (10 mg). A higher dose (50 mg) increased the incidence of false-positive lesions, and detectability was too low with use of a lower dose (2 mg) in this study^21^.

It is important to validate the results from the phase I study with the optimized dosage^22,23^. The aims of this confirmatory trial were to assess the reproducibility of the previous results on safety and feasibility of SPECT/CT, intraoperative radio detection, and NIRF-guided surgery after intravenous administration of the optimal dose of 10 mg ^111^In-labelled DOTA–labetuzumab-IRDye800CW for the detection and treatment of peritoneal metastases of colorectal origin before surgery.

## Methods

A single-centre, open-label confirmatory cohort phase II study (NCT03699332) was conducted to evaluate the safety and feasibility of image-guided surgery using ^111^In-labelled DOTA–labetuzumab-IRDye800CW.

The preparation of ^111^In-labelled DOTA–labetuzumab-IRDye800CW was described previously^21^. Briefly, labetuzumab (Research Resource Identifier AB_2910852) was first incubated with IRDye800CW-*N*-hydroxysuccinimide (NHS) and DOTA-NHS. After conjugation, the product was purified via dialysis, sterile filtrated, aliquoted, and stored. On the day of injection, DOTA–labetuzumab-IRDye800CW was radiolabelled with ^111^In (100 MBq).

### Subject selection

Patients aged ≥ 18 years with confirmed peritoneal metastases of colorectal cancer scheduled for CRS-HIPEC or redo CRS-HIPEC were selected for inclusion. Exclusion criteria were: pregnant or breastfeeding women; patients with a serum CEA concentration > 500 ng/ml; a known CEA-negative malignancy; recent administration of a radionuclide no later than 10 physical half-lives before study enrolment; or the presence of other uncontrolled medical conditions potentially jeopardizing the patients’ well-being and/or the study. The trial was approved by the regional ethical review board MREC Oost-Nederland, formerly known as Committee on Research Involving Human Subjects region Arnhem–Nijmegen, adhering to the principles of the Declaration of Helsinki. Before participation, written informed consent was obtained from all patients.

### Safety

Patients received a single intravenous dose of ^111^In-labelled DOTA–labetuzumab-IRDye800CW 5 or 6 days before surgery and were continuously monitored for adverse events (AEs) up to 3 hours after injection. AEs associated with the study procedures, not necessarily linked to ^111^In-labelled DOTA–labetuzumab-IRDye800CW administration, were considered treatment-related if they occurred within 10 days of surgery, in accordance with the National Cancer Institute Common Terminology Criteria for AEs (version 4.03).

### SPECT/CT before surgery

This was conducted 4 or 5 days following the administration of the dual-labelled antibody to obtain images of the thorax and abdomen. A physician specializing in nuclear medicine assessed the accumulation of ^111^In-labelled DOTA–labetuzumab-IRDye800CW using the PCI score.

### Image-guided surgery using NIRF camera and γ probe

Following surgical exposure and examination of the abdomen, the PCI was evaluated using standard methods. Subsequently, fluorescence-based PCI assessment was conducted using a Spectrum^®^ NIRF camera (Quest Medical Imaging, Middenmeer, the Netherlands). Radiosignal counts for background measurements were taken from the liver, abdominal wall, and colon. Cytoreductive surgery was then carried out in accordance with usual practice, including the excision of any additional lesions detected with NIRF imaging or increased radiosignal.

After complete cytoreduction, exploration of the abdominal cavity was carried out using both the Europrobe^©^ 3.2 SOE 3216-7 (Eurorad SA, Eckbolsheim, France) and the Spectrum^®^ NIRF camera for remnant tumour tissue. Radiodetection and NIRF imaging were conducted on all resected tissue specimens. Quantification of tracer accumulation was undertaken through back-table γ probe detection for 5 seconds on all suspected malignant lesions, with the highest result being registered for analysis. For each lesion, one corresponding benign region (background tissue) was used as a negative control.

### Tissue analysis

Tissue slides (4 µm) from all formalin-fixed paraffin-embedded blocks were scanned using an Odyssey^®^ CLx flatbed fluorescence imaging system (LI-COR Biosciences, Lincoln, NE, USA) at 800 nm for fluorescent signal detection. The images were then exported in tagged image file format using Image Studio™ Lite 5.2.5 (LI-COR Biosciences). After scanning, the sections were stained with haematoxylin and eosin and for CEA to confirm the presence of colorectal tumour cells. Following this confirmation, specific regions of interest (ROIs), delineating tumours and adjacent normal tissues, were identified on the haematoxylin and eosin slides using CaseViewer™ 2.4 (3Dhistech, Budapest, Hungary). These ROIs were subsequently imported into Fiji-ImageJ (Version 1.51) for quantifying mean fluorescence intensity in both tumour and benign tissues^24^.

### Study outcomes

The primary outcomes of this study were the safety and feasibility of multimodal image-guided surgery following the intravenous administration of ^111^In-labelled DOTA–labetuzumabIRDye800CW in patients with CRS-HIPEC with colorectal peritoneal carcinomatosis. The safety of image-guided surgery relied on the absence of severe AEs related to ^111^In-labelled DOTA–labetuzumab-IRDye800CW administration or study procedures. Feasibility was determined by detecting specific accumulation of ^111^In-labelled DOTA–labetuzumab-IRDye800CW in CEA-expressing tumour tissue using available technologies (radiodetection, optical imaging, and (immuno)histological analyses).

Secondary outcomes were identifying additional lesions via NIRF imaging after standard CRS, evaluating tumour-to-background ratios (TBRs) through *ex vivo* γ measurements of surgical specimens and flatbed fluorescence measurements of 4-µm tissue sections, and assessing the relationship between tracer accumulation and CEA expression. This study did not repeat dose assessments or pharmacokinetic analyses from the previous proof-of-concept study^21^.

### Statistical analysis

Descriptive statistics, including mean radiosignal and fluorescence-based TBRs, are depicted as mean(standard deviation, s.d.). SPECT/CT PCI and surgical PCI scores were depicted as mean(s.d.) and compared using a paired-samples Student’s *t* test. Statistical analyses were undertaken using GraphPad™ Prism version 5.03 (GraphPad Software, San Diego, CA, USA) and SPSS^®^ version 25.0 (IBM, Armonk, NY, USA). *P* < 0.050 was considered statistically significant.

## Results

### Patient characteristics and safety

**Fig. 1:**
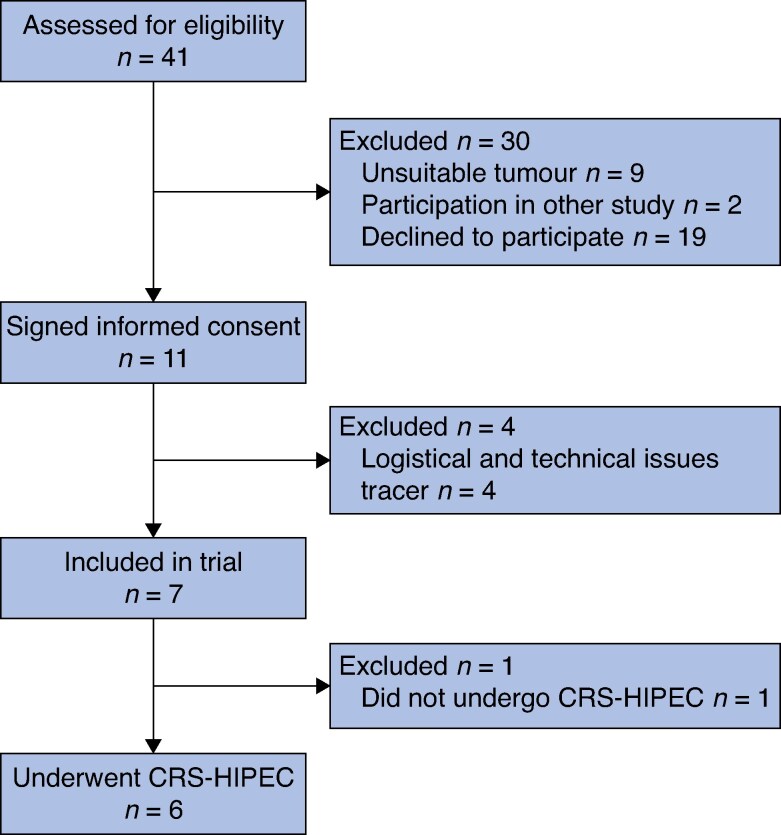
Flow diagram showing patient inclusion CRS, cytoreductive surgery; HIPEC, hyperthermic intraperitoneal chemotherapy.

A total of 41 patients were scheduled for cytoreductive surgery, of whom 9 had an unsuitable tumour on histological assessment. Eleven patients provided written informed consent for the study; four patients were excluded because of technical issues with tracer or equipment, leaving seven for assessment in this study (*Fig. 1*). Median age of the participants was 71 (range 48–82) years. Temperature, blood pressure, and haematological markers for liver and renal function remained stable in the hours after injection (day 0), at time of the SPECT/CT (day 4/5), and until the day of surgery (day 5/6). The mean ^111^In dose received was 101.25 (range 94.72–104.5) MBq. Median length of hospital stay was 14 (range 5–47) days (*Table 1*). No study-related AEs occurred according to National Cancer Institute Common Terminology Criteria; however, five severe AEs were reported, though none were related to study procedures. All reported AEs are summarized in *Table 2*.

**Table 1:** Clinical characteristics

Patient	Age (years), sex	Treatment before surgery	Pattern of metastasis	Histology	Completeness of cytoreduction score	Clinical PCI score	SPECT PCI score	Pathological PCI score	Length of hospital stay (days)
1	71, F	None	SM	AC	CC0	14	2	14	43
2	70, F	None	SM	MAC	CC0	14	3	3	14
3	48, F	None	MM	AC	CC0	21	4	21	11
4	78, M	None	MM	AC	CC0	15	4	15	14
5	82, M	None	SM	AC	CC0	3	2	3	24
6	73, M	Systemic therapy	SM	AC	CC0	6	0	4	47
7	50, F	None	SM	MAC	CC2	> 23	6	NA	5

PCI, peritoneal cancer index; SPECT, single-photon emission computed tomography; F, female; SM, synchronous metastases; AC, adenocarcinoma; MAC, mucinous adenocarcinoma; MM, metachronous metastases; M, male.

**Table 2:** AEs

Patient	Time after injection (days)	AE	Intervention	Related to study	CTCAE grade	Severe AE
1	11 12 13 16	Fever (without focus) Chyle leakage Gastroparesis Aspiration pneumonia	Antibiotics MCT diet Nasogastric tube Antibiotics	No No No No	1 1 3 3	No No No No
2	7	CVC line infection	Antibiotics	No	3	No
4	11	Subsegmental pulmonary embolism	Non-invasive ventilation	No	2	Yes
5	11 11 14 18	Multiple pulmonary embolism Ileus Jugular vein thrombus Fascial dehiscence	Enema Relaparotomy	No No No No	1 3 2 3	No No No Yes
6	14 18 25 27	Anastomotic leakage Wound infection Parastomal abscess Fascial dehiscence	Relaparotomy Antibiotics Drainage Relaparotomy	No No No No	3 3 1 3	Yes Yes Yes No

AE, adverse event; CTCAE, common terminology criteria for adverse events; MCT, medium chain triglycerides; CVC, central venous catheter.

### SPECT/CT before surgery

**Fig. 2:**
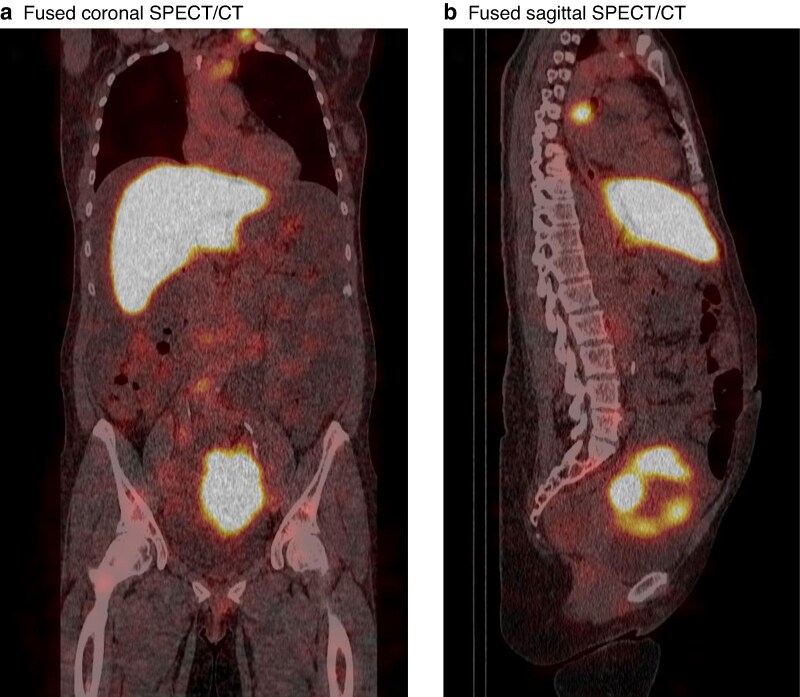
Multimodal imaging of previously undetected lymph node metastases (patient 7) **a** Retroclavicular node, **b** para-oesophageal node. SPECT, single-photon emission computed tomography; CT, computed tomography.

Six of seven patients underwent SPECT/CT 4 or 5 days after tracer injection. One patient did not undergo SPECT/CT because of suspected COVID-19 infection. SPECT/CT revealed peritoneal metastases in five of six patients. The mean(s.d.) SPECT/CT PCI score was 3(2). CEA-targeted SPECT/CT revealed lesions such as primary tumours, ovarian metastases, lymph node metastases, and some peritoneal metastases, all > 10 mm. In one patient, there were additional findings outside the abdominal cavity during SPECT/CT, which showed accumulation of tracer in retroclavicular and paraoesophageal nodes (*Fig. 2*). This patient also had extensive peritoneal disease during laparotomy with a PCI score > 23.

### Surgical procedure and histopathological analysis

All patients were primary candidates for CRS-HIPEC. Six patients underwent complete cytoreduction of peritoneal metastases. Two of these had synchronous metastasis and their primary tumours were resected in the same procedure. One patient did not undergo CRS-HIPEC because of a high clinical PCI score during exploratory laparotomy, resulting in termination of the procedure. The mean(s.d.) surgical PCI was 14(7), which was significantly higher than the mean SPECT/CT PCI score of 3(2) (*P* =0.032). The mean pathological PCI score was 9(8).

In total, 52 lesions were removed for pathological assessment and measured by NIRF imaging. Final pathology demonstrated that 37 of these were malignant, of which 34 (92%) were detected with NIRF imaging. Four tumour-positive lesions were not detected by NIRF imaging. Four of the 52 lesions (8%) were found to be histopathologically benign but were falsely assessed as positive for fluorescence by the surgeon. All these false-positive lesions were found in one patient, and pathology revealed three granulomas and a diverticulum.

**Fig. 3:**
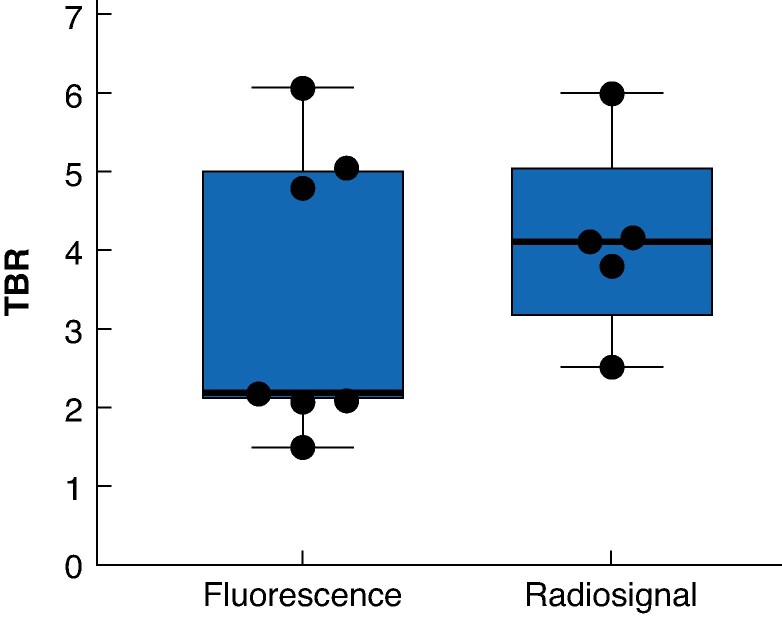
Mean fluorescence and mean radiosignal TBR per patient Black dots indicate values for individual patients. Median value (bold line), interquartile range (box), and range (error bars) are also shown. TBR, tumour-to-background ratio.

Back-table γ counting was undertaken in five of seven patients because of technical issues with the probe in two patients. In 48 of the 52 resected lesions, γ counting was undertaken. This revealed a mean(s.d.) TBR of 4.4(1.4) in all malignant lesions (*Fig. 3*). Three of the four false-positive fluorescent lesions emitted a low radiosignal, with a mean TBR of 1.3(0.5). One of the false-positive fluorescent lesions had a radiosignal TBR of 2.7.

A total of 65 histological slides from the 52 lesions were included in the final analysis, and all slides exhibited clear overlap of the NIRF signal and localization of CEA-expressing tumour cells. Fluorescence TBR was 3.4(1.8) (*Fig. 3*). Further detailed fluorescent and radioactive measurements are provided in the *supplementary material*.

**Fig. 4:**
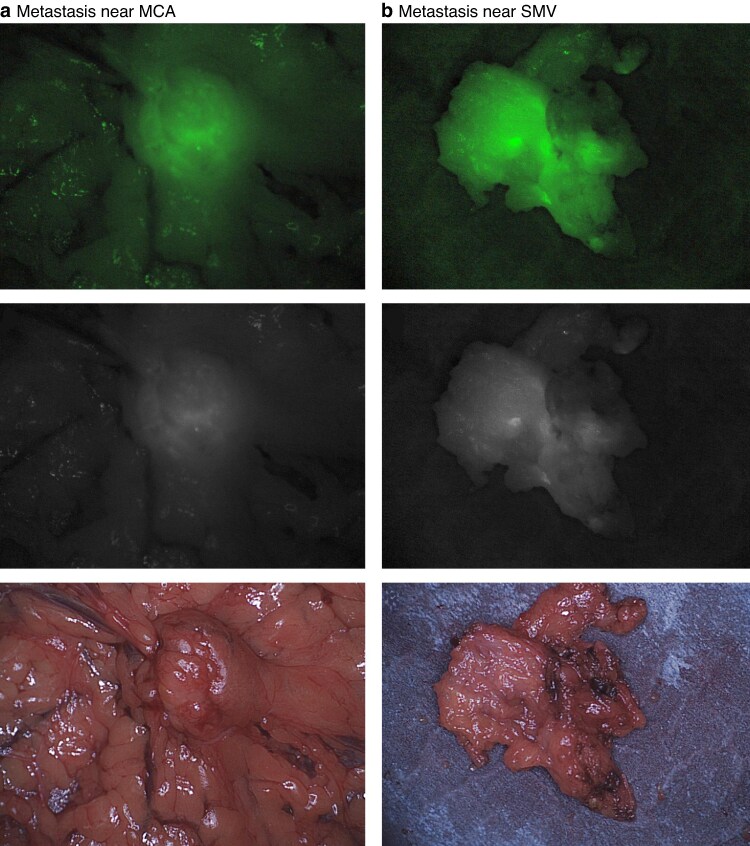
*In vivo* and *ex vivo* fluorescence detection in peritoneal metastases (patient 1) **a** Intraoperative near-infrared fluorescence (NIRF) imaging of a metastatic node near the medial colic artery (MCA), **b**  *ex vivo* NIRF imaging of suspicious node near the superior mesenteric vein (SMV). From top to bottom: NIR-fluorescence colour overlay, NIR-fluorescence, brightfield.

Multimodal image-guided surgery resulted in alteration of the clinical strategy in three of seven patients. A total of five lesions were missed initially during surgical inspection, but were identified by NIRF imaging, resulting in additional resections of NIRF-positive lesions (*Fig. 4*).

## Discussion

In this confirmatory trial, the optimal dose of 10 mg ^111^In-labelled DOTA–labetuzumab-IRDye800CW, as established in a previous phase I dose-finding study^21^, was administered to seven patients. Intravenous administration of the compound was safe and well tolerated, with no AEs reported during the study period. Tumour-targeted intraoperative NIRF imaging and radioguidance enabled successful identification, localization, and delineation of colorectal tumours, lymph node metastases, and peritoneal metastases. These findings were supported by quantitative analyses of TBRs for both fluorescence and radiosignals conducted *ex vivo*. Five additional lesions that were initially missed by routine abdominal evaluation during surgery were detected with intraoperative NIRF imaging. In three patients, this resulted in additional surgical resections. This further supports the value of intraoperative NIRF imaging and radiodetection as shown in the previous study^21^.

During surgery, NIRF imaging confirmed 92% of the malignant lesions, which was comparable to the 16 malignant lesions (95%) found in the previously described five patients with the 10-mg dose^21^. A similar study^25^ including patients undergoing HIPEC was undertaken, using a single-labelled fluorescent tracer, that showed a sensitivity of 98.5%. This small discrepancy in sensitivity can perhaps be explained by the experience of the surgical team with the fluorescence camera or different tumour locations. Of the 52 excised lesions, four (8%) were false-positive on NIRF imaging; this was also comparable to the previous 10-mg cohort, which showed three lesions (16%) as false-positive^21^. A possible explanation could be that differentiating genuine positive fluorescence from autofluorescence or non-tumour-related causes is not consistently feasible, as shown in previous studies^16,26,27^. Three lesions showed low tracer uptake (mean TBR 1.3), confirming the additional significance of the radiosignal. Multimodal imaging using a dual-labelled tracer is therefore a promising technique whereby radionuclide detection is complementary to NIRF imaging. Fluorescence signals can be detected with high spatial accuracy, but only superficially, in contrast to radionuclide detection, which makes it possible to examine deeper tissues.

Imaging with CEA-targeted SPECT/CT before surgery did not contribute to a more reliable PCI assessment, but revealed a contraindication to CRS-HIPEC by detecting retroclavicular and paraoesophageal lymph nodes in one patient that were missed on standard imaging. This is clinically significant because patients with peritoneal metastases and extraperitoneal/systemic disease are usually not eligible for CRS-HIPEC. CEA-targeted SPECT/CT could be used to avoid futile laparotomies and associated morbidity in the future, serving as a potentially valuable diagnostic tool with an impact on treatment policies. In the future, it is likely that CT scanners will have increased resolution and speed that enable visualization of even smaller anatomical structures and abnormalities, resulting in a higher sensitivity for this tracer and maximizing the impact of dual-labelled tracers^28^. This could also be applicable to other CEA-expressing malignancies, such as oesophageal and pancreatic cancers^26,29–31^.

The authors are currently studying whether multimodal imaging can be applied to patients with rectal and pancreatic cancer, using another multimodal ^111^In-labelled DOTA–anti-CEA antibody (NCT06395337). The principle of multimodal imaging using dual-labelled antibodies is a promising research development that extends beyond gastrointestinal malignancies. For example, ^111^In-labelled DOTA–girentuximab-IRDye800CW, a carbonic anhydrase IX-targeting antibody previously used in clear cell renal cell carcinoma^19^, and the monoclonal antibody D2B^32^ named ^111^In-labelled prostate-specific membrane antigen-IRDye800CW, which has been used in prostate carcinoma, have both seen promising results^32^. Studies with fluorescently labelled antibodies (without a radiolabel), such as SGM-101, have also been shown to be feasible^18,26,33^. Currently, a multicentre phase II–III study is ongoing in the Netherlands using SGM-101 in locally advanced and recurrent rectal cancer (NCT04642924).

Complete surgical resection remains fundamental in CRS-HIPEC treatment in patients with metastatic colorectal disease^34^; therefore, clinicians will persistently encounter significant procedure-related morbidity while undertaking extensive resections^35,36^. Intraoperative imaging appears to be a promising technique in supporting the surgeon in targeted resection. Previous studies have already demonstrated its potential value to patients with colorectal metastases. Boogerd *et al*.^16^ used a fluorescent anti-CEA tracer in patients with both primary and metastatic colorectal cancer and demonstrated alterations of surgical strategy based on intraoperative fluorescence imaging in 35% of patients. Combining the study results of de Gooyer *et al*.^21^ and this present study, 12 patients in total have received 10 mg ^111^In-labelled DOTA–labetuzumab-IRDye800CW. This resulted in a change of clinical management in six patients by additional resection of suspected lesions based on intraoperative imaging. Furthermore, extraperitoneal disease was demonstrated in two patients; this had a potentially significant impact, as a major procedure with futile advantage for the patient could have been avoided.

Four false-negative non-fluorescent lesions were detected, of which three were in one patient. These lesions were deemed malignant after γ counting, with a mean TBR of 2.5(0.49), and pathological analysis. Of all 46 clinically suspicious lesions 11 (24%) did not contain malignant cells, supporting the overestimation of clinical PCI^37^. The specificity and negative predictive value of multimodal imaging needs confirmation in a broader study population. Nevertheless, these findings suggest that, in the future, surgeons might opt to leave non-fluorescent lesions *in situ* if no raised radiosignal is detected, potentially resulting in more precise cytoreduction and reduced surgical trauma.

## Supplementary Material

zraf045_Supplementary_Data

## Data Availability

This study is a replication of a previously published study. Because of patient privacy constraints, the data produced during this trial are not publicly accessible. However, they can be obtained from the corresponding author upon reasonable request and approval from the trial sponsor, following the applicable guidelines at the time of the request.
