# Supplementary material for: Multimodal carcinoembryonic antigen-targeted fluorescence and radio-guided cytoreductive surgery for peritoneal metastases of colorectal origin: single-arm confirmatory trial
Source: BJS Open. 2025 Apr 24;9(2):zraf045. doi: 10.1093/bjsopen/zraf045 (PMC12018875; doi:10.1093/bjsopen/zraf045)
Supplement: zraf045_Supplementary_Data [file zraf045_supplementary_data.docx]

Multimodal CEA-targeted fluorescence and radio guided cytoreductive surgery for peritoneal metastases of colorectal origin: a single-arm confirmatory trial

Aaya Darai^a^, Jan Marie de Gooyer^ac^, Sander Ubels^a^, Andreas J.A. Bremers^a^, Philip R. de Reuver^a^, Erik H.J.G. Aarntzen^c^, Iris D. Nagtegaal^b^, Mark Rijpkema^c^, Johannes H.W. de Wilt^a^

1. Department of Surgery, Radboud University Medical Centre, Nijmegen, The Netherlands
2. Department of Pathology, Radboud University Medical Centre, Nijmegen, The Netherlands
3. Department of Medical imaging, Nuclear medicine, Radboud University Medical Centre, Nijmegen, Netherlands

**Corresponding author:**

Drs. Aaya Darai M.D.

Aaya.darai@radboudumc.nl

+31634251046

<https://orcid.org/0009-0000-6138-6398>

Department of Surgery

Radboud University Medical Center

Geert Grooteplein Zuid 10

6525GA Nijmegen, The Netherlands

**Supplementary Materials - Index**

| **Supplementary Figures and Tables** |  |
| --- | --- |
| **Table 1. Fluorescent and radioactive lesions** | *page 2* |
| **Table 2. Fluorescence and radiosignal of malignant tissue, per patient** | *page 2* |
| **References** | *page 3* |
|  |  |

**Supplementary Figures and Tables**

**Table 1. Fluorescent and radioactive lesions**

|  | **Malignant** | **Benign** |
| --- | --- | --- |
| **Fluorescence +**  **Fluorescence -** | 34 | 4 |
|  | 4 | 10 |
| ***Radioactive +**  **Radioactive -** | 30 | 2 |
|  | 4 | 12 |

****For four lesions radiodetection was not performed***

**Table 2. Fluorescence and radiosignal of malignant tissue, per patient**

| Patient | mean fluorescence signal | min-max | mean background | min-max background | mean TBR | mean radiosignal counts | min-max | mean background | min-max background | mean TBR |
| --- | --- | --- | --- | --- | --- | --- | --- | --- | --- | --- |
| 1 | 21.0 | 18.9-24.3 | 9.6 | 9.5-9.8 | **2.2** | 236 | 54-767 | 43 | 14-80 | **6.0** |
| 2 | 21.9 | 17.6-28.4 | 10.5 | 10.2-10.8 | **2.1** | 194 | 83-270 | 75 | 46-70 | **2.5** |
| 3 | 16.4 | 14.7-17.1 | 11.0 | 10.7-11.2 | **1.5** | 386 | 70-883 | 97 | 24-173 | **3.8** |
| 4 | 20.0 | 15.4-23.0 | 9.6 | 9.4-9.9 | **2.1** | 119 | 2-317 | 25 | 02-66 | **4.1** |
| 5 | 23.0 | 13.6-33.1 | 4.6 | 4.4-4.7 | **5.1** | NA | NA | NA | NA | **NA** |
| 6 | 21.9 | 15.7-28.0 | 4.6 | 4.3-4.6 | **4.8** | 89 | 40-182 | 22 | 16-30 | **4.2** |
| 7 | 26.8 | 16.4-49.3 | 4.5 | 4.4-4.6 | **6.1** | NA | NA | NA | NA | **NA** |

**References**

1. Rijken, A., et al., *Primary tumor resection or systemic treatment as palliative treatment for patients with isolated synchronous colorectal cancer peritoneal metastases in a nationwide cohort study.* Clin Exp Metastasis, 2023. **40**(4): p. 289-298.

2. van Gestel, Y.R., et al., *Metachronous peritoneal carcinomatosis after curative treatment of colorectal cancer.* Eur J Surg Oncol, 2014. **40**(8): p. 963-9.

3. Razenberg, L.G., et al., *Challenging the dogma of colorectal peritoneal metastases as an untreatable condition: Results of a population-based study.* Eur J Cancer, 2016. **65**: p. 113-20.

4. Quere, P., et al., *Epidemiology, Management, and Survival of Peritoneal Carcinomatosis from Colorectal Cancer: A Population-Based Study.* Dis Colon Rectum, 2015. **58**(8): p. 743-52.

5. Verwaal, V.J., et al., *8-Year Follow-up of Randomized Trial: Cytoreduction and Hyperthermic Intraperitoneal Chemotherapy Versus Systemic Chemotherapy in Patients with Peritoneal Carcinomatosis of Colorectal Cancer.* Annals of Surgical Oncology, 2008. **15**(9): p. 2426-2432.

6. Kuijpers, A.M., et al., *Cytoreduction and HIPEC in the Netherlands: nationwide long-term outcome following the Dutch protocol.* Ann Surg Oncol, 2013. **20**(13): p. 4224-30.

7. Elias, D., et al., *Peritoneal colorectal carcinomatosis treated with surgery and perioperative intraperitoneal chemotherapy: retrospective analysis of 523 patients from a multicentric French study.* J Clin Oncol, 2010. **28**(1): p. 63-8.

8. van der Ven, R., et al., *Towards Equal Access to Cytoreductive Surgery with Hyperthermic Intraperitoneal Chemotherapy and Survival in Patients with Isolated Colorectal Peritoneal Metastases: A Nationwide Population-Based Study.* Ann Surg Oncol, 2024.

9. Hallam, S., et al., *Meta-analysis of prognostic factors for patients with colorectal peritoneal metastasis undergoing cytoreductive surgery and heated intraperitoneal chemotherapy.* BJS Open, 2019. **3**(5): p. 585-594.

10. van Oudheusden, T.R., et al., *Peritoneal cancer patients not suitable for cytoreductive surgery and HIPEC during explorative surgery: risk factors, treatment options, and prognosis.* Ann Surg Oncol, 2015. **22**(4): p. 1236-42.

11. Jacquet, P. and P.H. Sugarbaker, *Clinical research methodologies in diagnosis and staging of patients with peritoneal carcinomatosis.* Cancer Treat Res, 1996. **82**: p. 359-74.

12. Rivard, J.D., et al., *Preoperative computed tomography does not predict resectability in peritoneal carcinomatosis.* Am J Surg, 2014. **207**(5): p. 760-4; discussion 764-5.

13. Laghi, A., et al., *Diagnostic performance of computed tomography and magnetic resonance imaging for detecting peritoneal metastases: systematic review and meta-analysis.* Radiol Med, 2017. **122**(1): p. 1-15.

14. Elekonawo, F.M.K., et al., *Can [18F]F-FDG PET/CT be used to assess the pre-operative extent of peritoneal carcinomatosis in patients with colorectal cancer?* Abdominal Radiology, 2020. **45**.

15. Rijpkema, M., et al., *SPECT- and fluorescence image-guided surgery using a dual-labeled carcinoembryonic antigen-targeting antibody.* J Nucl Med, 2014. **55**(9): p. 1519-24.

16. Boogerd, L.S.F., et al., *Safety and effectiveness of SGM-101, a fluorescent antibody targeting carcinoembryonic antigen, for intraoperative detection of colorectal cancer: a dose-escalation pilot study.* Lancet Gastroenterol Hepatol, 2018. **3**(3): p. 181-191.

17. Harlaar, N.J., et al., *Molecular fluorescence-guided surgery of peritoneal carcinomatosis of colorectal origin: a single-centre feasibility study.* Lancet Gastroenterol Hepatol, 2016. **1**(4): p. 283-290.

18. de Gooyer, J.M., et al., *Multimodal CEA-Targeted Image-Guided Colorectal Cancer Surgery using (111)In-Labeled SGM-101.* Clin Cancer Res, 2020. **26**(22): p. 5934-5942.

19. Hekman, M.C., et al., *Tumor-targeted Dual-modality Imaging to Improve Intraoperative Visualization of Clear Cell Renal Cell Carcinoma: A First in Man Study.* Theranostics, 2018. **8**(8): p. 2161-2170.

20. van Dam, G.M., et al., *Intraoperative tumor-specific fluorescence imaging in ovarian cancer by folate receptor-α targeting: first in-human results.* Nature Medicine, 2011. **17**(10): p. 1315-1319.

21. de Gooyer, J.M., et al., *Multimodal CEA-targeted fluorescence and radioguided cytoreductive surgery for peritoneal metastases of colorectal origin.* Nature Communications, 2022. **13**(1): p. 2621.

22. Viera, C., *Why is Replication in Research Important?* American Journal of Experts, 2023.

23. Raphael M.P., S.P.E., Vora G.J., *A controlled trial for reproducibility.* Nature, 2020. **579**: p. 190-192.

24. Schaap, D.P., et al., *Carcinoembryonic antigen-specific, fluorescent image-guided cytoreductive surgery with hyperthermic intraperitoneal chemotherapy for metastatic colorectal cancer.* Br J Surg, 2020. **107**(4): p. 334-337.

25. Meijer, R.P.J., et al., *Intraoperative detection of colorectal and pancreatic liver metastases using SGM-101, a fluorescent antibody targeting CEA.* Eur J Surg Oncol, 2021. **47**(3 Pt B): p. 667-673.

26. Zhou, Q., et al., *Factors for Differential Outcome Across Cancers in Clinical Molecule-Targeted Fluorescence Imaging.* J Nucl Med, 2022. **63**(11): p. 1693-1700.

27. Estimates, G.M. *Next-Gen CT Scanners: What to Expect 2028*. 2023 21-12-2023 16-05-2024]; Available from: <https://www.globalmarketestimates.com/blogpost/next-gen-ct-scanners-what-to-expect-by-2028-181#:~:text=Advanced%20CT%20Imaging%20Technology,detection%20of%20disease%20or%20abnormalities>.

28. Shi, N.Q., et al., *Application of near-infrared fluorescence imaging in theranostics of gastrointestinal tumors.* Gastroenterol Rep (Oxf), 2023. **11**: p. goad055.

29. Wong, P., et al., *Antibody Targeted PET Imaging of (64)Cu-DOTA-Anti-CEA PEGylated Lipid Nanodiscs in CEA Positive Tumors.* Bioconjug Chem, 2020. **31**(3): p. 743-753.

30. van Dam, M.A., et al., *Overview and Future Perspectives on Tumor-Targeted Positron Emission Tomography and Fluorescence Imaging of Pancreatic Cancer in the Era of Neoadjuvant Therapy.* Cancers (Basel), 2021. **13**(23).

31. Lütje, S., et al., *Dual-Modality Image-Guided Surgery of Prostate Cancer with a Radiolabeled Fluorescent Anti-PSMA Monoclonal Antibody.* J Nucl Med, 2014. **55**(6): p. 995-1001.

32. de Valk, K.S., et al., *Dose-Finding Study of a CEA-Targeting Agent, SGM-101, for Intraoperative Fluorescence Imaging of Colorectal Cancer.* Ann Surg Oncol, 2021. **28**(3): p. 1832-1844.

33. Quénet, F., et al., *Cytoreductive surgery plus hyperthermic intraperitoneal chemotherapy versus cytoreductive surgery alone for colorectal peritoneal metastases (PRODIGE 7): a multicentre, randomised, open-label, phase 3 trial.* Lancet Oncol, 2021. **22**(2): p. 256-266.

34. Kuijpers, A.M., et al., *Implementation of a standardized HIPEC protocol improves outcome for peritoneal malignancy.* World J Surg, 2015. **39**(2): p. 453-60.

35. Ammerata, G., et al., *Hyperthermic intraperitoneal chemotherapy and colorectal cancer: From physiology to surgery.* World J Clin Cases, 2022. **10**(30): p. 10852-10861.

36. de Boer, N.L., et al., *The Accuracy of the Surgical Peritoneal Cancer Index in Patients with Peritoneal Metastases of Colorectal Cancer.* Dig Surg, 2021. **38**(3): p. 205-211.
